# Simulation study and comparative evaluation of viral contiguous sequence identification tools

**DOI:** 10.1186/s12859-021-04242-0

**Published:** 2021-06-16

**Authors:** Cody Glickman, Jo Hendrix, Michael Strong

**Affiliations:** 1 Center for Genes, Environment, and Health, National Jewish Health, 1400 Jackson Street, Denver, CO 80206 USA; 2 Computational Bioscience, University of Colorado Anschutz, 12801 E 17th Avenue, Aurora, CO 80045 USA

**Keywords:** Virus, Bacteriophage, Prophage, Metagenomics, Tool comparison

## Abstract

**Background:**

Viruses, including bacteriophages, are important components of environmental and human associated microbial communities. Viruses can act as extracellular reservoirs of bacterial genes, can mediate microbiome dynamics, and can influence the virulence of clinical pathogens. Various targeted metagenomic analysis techniques detect viral sequences, but these methods often exclude large and genome integrated viruses. In this study, we evaluate and compare the ability of nine state-of-the-art bioinformatic tools, including Vibrant, VirSorter, VirSorter2, VirFinder, DeepVirFinder, MetaPhinder, Kraken 2, Phybrid, and a BLAST search using identified proteins from the Earth Virome Pipeline to identify viral contiguous sequences (contigs) across simulated metagenomes with different read distributions, taxonomic compositions, and complexities.

**Results:**

Of the tools tested in this study, VirSorter achieved the best F1 score while Vibrant had the highest average F1 score at predicting integrated prophages. Though less balanced in its precision and recall, Kraken2 had the highest average precision by a substantial margin. We introduced the machine learning tool, Phybrid, which demonstrated an improvement in average F1 score over tools such as MetaPhinder. The tool utilizes machine learning with both gene content and nucleotide features. The addition of nucleotide features improves the precision and recall compared to the gene content features alone.Viral identification by all tools was not impacted by underlying read distribution but did improve with contig length. Tool performance was inversely related to taxonomic complexity and varied by the phage host. For instance, *Rhizobium* and *Enterococcus* phages were identified consistently by the tools; whereas, *Neisseria* prophage sequences were commonly missed in this study.

**Conclusion:**

This study benchmarked the performance of nine state-of-the-art bioinformatic tools to identify viral contigs across different simulation conditions. This study explored the ability of the tools to identify integrated prophage elements traditionally excluded from targeted sequencing approaches. Our comprehensive analysis of viral identification tools to assess their performance in a variety of situations provides valuable insights to viral researchers looking to mine viral elements from publicly available metagenomic data.

**Supplementary Information:**

The online version contains supplementary material available at 10.1186/s12859-021-04242-0.

## Background

Viruses are the most abundant biological entities on Earth [1]. However, the collective knowledge of environmental viral sequences, including bacteriophages, remains underrepresented relative to the amount of genetic information for eukaryotic viruses and bacteria. Bacteriophages are viruses that infect bacteria and are commonly referred to as phages. Phages are obligate parasites that play an important role in the genomic composition and evolution of their bacterial hosts. Phages directly contribute to bacterial infections in humans by acting as a genetic reservoir for virulent genes in bacteria such as *Escherichia coli*, *Salmonella enterica*, *Pseudomonas aeruginosa*, *Vibrio cholerae*, *Corynebacterium diphtheriae*, and *Streptococcus pyogenes* [2, 3].

In addition, some phages utilize Ig-like domains to attach to mucosal layers in humans to lie in wait for bacterial prey. This bacteriophage adherence to mucus (BAM) model suggests that phages may act as a non-host derived innate immunity system to modulate the bacterial microbiome [4]. A longitudinal study of the human virome revealed composition conservation that mimicked the stability of healthy bacterial microbiomes [5, 6]. Dysbiosis in the virome has been observed in disease states such as inflammatory bowel disease (IBD), Crohn’s disease, and asthma [7–9].

The study of viruses has traditionally relied on the ability to cultivate viral particles from a cultured host; however, many bacteria cannot be cultured in a laboratory setting [10]. The limited number of culturable hosts, in combination with the additional complexities of viral isolation limit the study of viruses. The advancements in next generation sequencing technologies created an opportunity to study viruses with culture independent methods. However, because viruses do not share a common universal marker gene, like the bacterial small subunit RNA, sequencing techniques such as metagenomics are a necessity [11]. Metagenomics is a non-targeted sequencing approach to elucidate the totality of genetic material within a sample, either DNA or RNA. However, in part due to small genomes, viruses are traditionally underrepresented in metagenomic studies from a read abundance perspective. It is common for viral reads to comprise less than 5% of metagenomic sequences [12]. A way to enrich viral reads in metagenomic studies is to filter or directly select viral like particles (VLPs). However, these techniques tend to remove large viruses and viruses integrated into bacterial genomes called prophages, before sequencing. Therefore, the ability to identify viral elements directly from metagenomic sequencing studies is also important for understanding the composition of the virome. The advent of computational tools dedicated to the identification of viral sequences in metagenomics has improved our ability to identify known, novel, and integrated viruses.

MetaPhinder is an approach that uses BLASTn and average nucleotide identity thresholds to classify viral contigs in metagenomics [13]. Methods that use sequence similarity suffers worsening performance with smaller contig lengths. Domain recognition is utilized by more tools to counter the limitations of contig length on traditional sequence homology approaches, but these tools are often reliant on specialized viral domains like those from pVOGs (prokaryotic virus orthologous groups) [14]. Unlike prophage identification methods that use viral domain enrichment or presence/absence to calculate a score, a new method, called Vibrant, uses domain abundances in a neural network framework to classify contigs having more than 4 proteins [15]. VirSorter2 follows a similar methodology, using domain percentages, gene content features, and key homology genes in a tree-based machine learning framework to classify viral reads [16].

Homology of viral protein domains is limited to known viruses, which are thought to represent only a small slice of the vast viral dark matter [17]. Another homology approach sought to expand known viral hidden Markov models (HMMs) through a semi-supervised expansion of existing viral protein families. Paez-Espino et al. (Earth Virome Pipeline) collected viral coding regions from NCBI servers and known viral metagenomic contigs; then clustered those peptides into protein families to create new viral HMMs [18]. This initial set was used as bait to identify potential viral contigs in thousands of metagenomic data sets. Predicted proteins from these captured viral contigs were added to the original set of peptides and re-clustered to create thousands of new viral protein families and HMMs. Even with the expansion of viral families, both VirSorter and the BLASTp search using the Earth Virome protein set are at least partially reliant on domain homology. A reference-free viral identification tool was developed using machine learning to address limitations of homology searching. VirFinder is a logistic regression classification using nucleotide sequence 8-mers as features [19]. The authors of VirFinder expanded the concept of using k-mers as features to identify viral contiguous sequences with DeepVirFinder, a convolutional neural network that takes raw sequences as inputs and learns features that are useful for viral contig prediction [20]. VirFinder relies solely on sequence-based features, which is analogous to another k-mer approach, Kraken2. Kraken2 uses discriminatory 35-mers to uniquely identify sequences to the species and even subspecies level [21]. In order to use Kraken2 in a viral identification context, we created the tool, VirKraken, that parses the Kraken2 classification output to assign viral contigs in metagenomic reads. VirKraken is available on PyPI and at https://github.com/Strong-Lab/VirKraken. VirKraken references the Kraken2 assigned taxonomy identification number against an edited NCBI Taxonomy database to assign kingdom and to filter sequences when requested [22].

Another approach to identify viral elements in metagenomics involves negation of known bacteria contigs. VirMine uses a homology search against a bacterial protein database; if hits of bacterial genes outnumber the number of unknown hits the contig is removed, thus leaving viral contigs [23]. All previously described tools identify viral elements from assembled sequences. MARVEL is a machine learning method that classifies binned contigs as viral clusters using a random forest approach with three features (gene density, strand shifts, and fraction of homology hits to a viral protein database) [24].

The authors of VirFinder put forth a call to create a hybrid tool that utilizes both k-mer features and gene content features to offset the weaknesses of both methods [19]. To answer that call, we developed a machine learning model called Phybrid that uses both gene content features such as gene density and strand shift frequency, in addition to sequence-based features to classify viral contigs using an additive boosting model. The addition of gene content features is hypothesized to offset the dip in performance of sequence-based machine learning models compared to homology methods on longer contigs [19].

Many approaches exist to identify viral elements in metagenomics. However, a systemic evaluation among many of these tools has not been performed. This study is meant to provide information and guidance to researchers regarding when to use a specific viral identification tool to further study viral elements or to remove them for downstream analyses. The characterization of more viral elements in the public domain could lead to the discovery of novel viruses [25] and provide insight into the functional potential residing in an extracellular genetic reservoir [2].

## Methods

### Phybrid, a hybrid gene content and nucleotide feature set for viral classification

To build Phybrid, 1849 complete phage and 2327 complete archaeal/bacterial genomes were compiled from RefSeq (Accessed on January 8th, 2020). Prophage elements in the archaeal and bacterial genomes were identified using VirSorter [22]. Category 4 prophages were selected, and the predicted nucleotide sequences were added to the complete viral genomes. Custom scripts were used to identify and remove the predicted prophage sequences from the host genome. The total number of prophages predicted was 730 in 339 bacterial genomes (14.57% of genomes contained at least 1 prophage) culminating in an average prophage per genome ratio of 0.314.

After removing integrated prophages, the complete genomes were fragmented into k-mers of 4 sizes using an n-step kmerization method. The n-step method removes contig end-overlap and ensures that the maximum number of k-mers is the length of the base sequence over the length k. The complete genomes were fragmented into sizes of 1 KB, 3 KB, 5 KB, and 10 KB sequences. Due to the size of bacterial and archaeal genomes relative to phage genomes, the fragments from non-phage sampling were down sampled to evenly distribute the classes. The four different fragment lengths were used to train four separate models.

The n-step fragments were subjected to a sliding window kmerization of size 8 using a k-mer counting program written in C [26]. A sliding window kmerization calculates k-mer abundance with significant overlap and the maximum number of k-mers is the length of the base sequences minus 1. The program stores all 8-mer values (65,536 possible 8-mers) in a hash table. In real world metagenomic sampling, the directionality of a sequence fragment may be ambiguous. Therefore, similar to VirFinder [19], we developed custom scripts to sum complement, reverse, and reverse complement sequences thus reducing our feature space from 65,536 possible k-mers to 16,384 possible k-mers. The nucleotide feature space is further reduced to 888 k-mers using Gini importance or total decrease in node impurity above 0.001, which is a weighted probability of reaching a feature averaged over all trees in a random forest [27].

### Gene content feature set creation

**Fig. 1:**
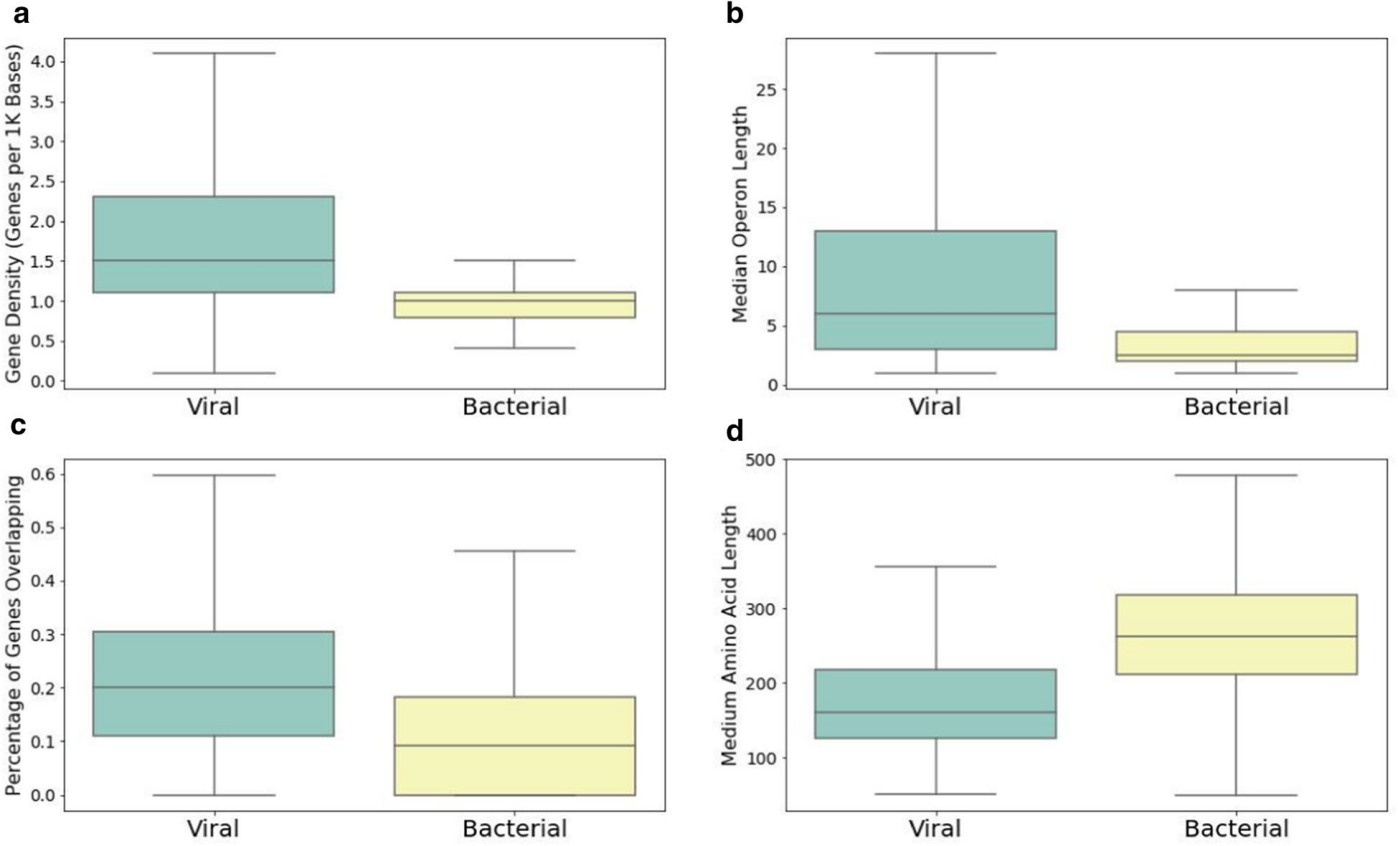
Gene content feature performance. The performance of the 4 gene content features in the 10 KB training dataset. **a** Gene density represented by number of genes per 1 KB. **b** Median operon length is a representative measure of strand switching frequency. An operon is defined as as a set of closely linked genes on the same strand. **c** Percentage of overlapping peptides measured as a percentage of all predicted genes. Viruses that have a lysogeny phase are known overlap genes for different life cycles. **d** Median amino acid length as viral peptides are commonly shorter than bacterial peptides

The use of gene content features are built into tools such as MARVEL and VirSorter [24, 28]. MARVEL and VirSorter both utilize gene density as a marker of viral elements. In this study, four gene content features associated with viral genomes were included as part of the feature set in Phybrid; gene density, operon length, average peptide length, and percentage of overlapping peptides. Due to the physical restraints of some viral capsids, viral genomes are commonly tightly packed and translate shorter proteins than bacterial genomes [29]. Operon length in the context of this study is the length of a continuous set of genes on the same strand. Viruses tend have long stretches of genes located on the same strand [30]. In addition, viral genomes often have overlapped genes for different life cycles [30]. Custom scripts were used to calculate the four protein characteristics from the output of the Prodigal gene prediction software [31]. Figure 1 shows the observed distribution of protein features in the training data of the 10 KB model.

### Model and hyperparameter selection

After combining the complementary nucleotide features and the gene content features, the total feature space of Phybrid was 892 features. During training, the performance of a random forest, multi-layer perceptron, and an additive boosting model were compared using 5-fold cross validation [32]. At every fragment size, the additive boosting model performed the best. We selected XGBoost (version 0.81) and performed a RandomSearchCV (version 0.20.1) analysis to determine hyperparameters [32, 33]. The pre-trained models were added to the tool repository for use classifying metagenomic fasta sequences. Phybrid generates outputs as a header file containing the header sequences of viral elements and a fasta file containing the nucleotide sequences of the predicted viral elements.

### Building simulated Illumina metagenomes

To build a simulated test set, all complete genomes were downloaded from NCBI RefSeq (accessed on 12/15/2020). The genomes deposited since May 1st, 2020, were selected to test the viral contig identification tools because many of the tools were trained or relied on databases last updated prior to this date. Bacterial hosts of phages were collected using a dataset from Virus-Host DB [34] (Accessed on December 17, 2020). Phage were assigned bacteria genera values by their host organism. Using information from the Earth Microbiome Project (EMP) and from Qiita, the recently submitted genomes were further filtered by 53 genera commonly found in soils (37 genera) and in clinical samples (26 genera) with 8 genera in both niches [35, 36]. This resulted in 297 unique bacterial genomes being used for the simulations with 82 genomes found in both clinical sampling (160 genomes) and soil sampling (219 genomes). The reliance on recently submitted genomes to produce the testing set did not produce traditional bacterial distributions seen in clinical and soil microbiomes. For example, while the genera *Bacteroides* are commonly present in the clinical microbe samples, the amount in this study does not represent a substantial portion of the community as seen in other clinical microbiome studies [37]. The distribution of bacterial genera was used as a confounder for viral classification in this study. The goal of this study was to observe the performance of phage identification in the presence of genetically similar bacteria.

Phage genomes were also filtered by their host bacterial genera and randomly down sampled to match the number of bacterial genomes in the simulations. While phages are thought to outnumber bacteria ten to one in the environment [38], we matched the complexity of phage and bacteria in our simulations across taxonomic levels due to limitations in the number of available phage genomes for the full datasets. In order to test the impact of taxonomic complexity on viral identification tool performance, we subsampled phage and bacterial genomes into medium (50 bacterial genomes and 50 phage genomes) and low (10 bacterial genomes and 10 phage genomes) complexity subsets. Additional file 2: Table S1 (clinical) and Additional file 2: Table S2 (soil) detail the taxonomic abundance of the top 6 genera and phage host genera in the testing set across taxonomic complexity levels. While both lower complexities draw from the full distribution of genomes, there is no overlap in the selected genomes between the medium and low taxonomic levels. This was accomplished through setting a random seed in the subsampling procedure and using set operations to confirm no overlap of genomes.

Simulated metagenomes were created using InSilicoSeq (version 1.2.0). InSilicoSeq and another popular metagenomic simulator, CAMISIM use a lognormal read distribution by default, however, four additional read distributions are provided as a part of the InSilicoSeq software suite: uniform, exponential, zero inflated lognormal, and halfnormal [39, 40]. Due to the enormous diversity of naturally occurring communities, read distribution profiles are likely to fluctuate. To understand the impact of read distribution and taxonomic complexities on the performance of viral identification, we created 30 MiSeq simulations with 12 million 2x300 reads. The 30 simulations were composed of two environmental conditions (clinical and soil microbes) with five read distributions across three taxonomic levels (full, medium, low). Bacterial reads represented 93.75% of the total composition in each simulation and phages represented 6.25%. Prior studies suggest phages commonly represent less than 5% of metagenomic sequencing reads [12] due to genomes that are orders of magnitude smaller than prokaryotic genomes. Our decision to exceed the 5% of viral reads in metagenomics was driven by the need to identify an expanded set of phages from taxonomically diverse testing sets. After assembly and filtration of contigs less than 1KB in length, phages comprise an average of 1.54% of total contig abundance.

**Fig. 2:**
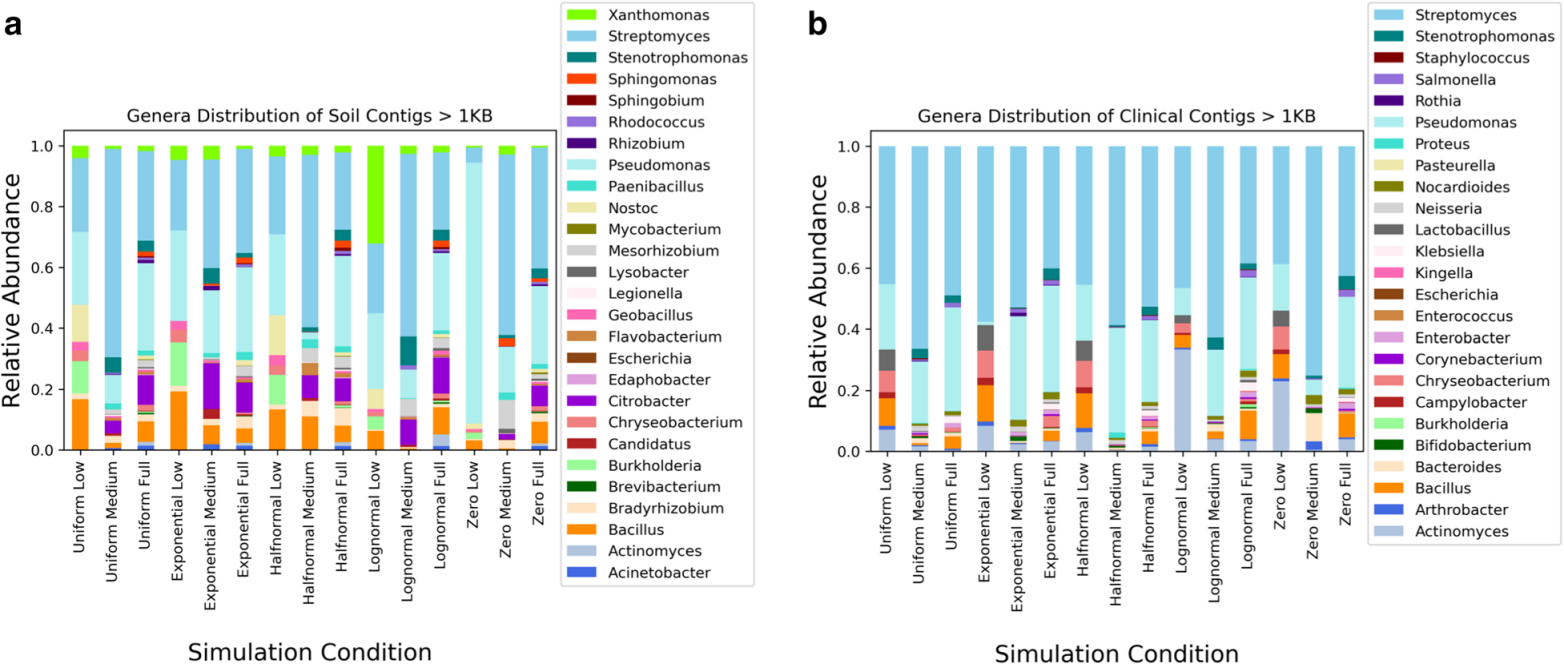
Relative abundance of genera in simulations. These figures highlight the relative abundance of the contigs greater than 1 KB. Bacterial contigs represent 98.46% of contigs, while phages and prophages combine for the remaining 1.54%. **a** The contig distribution within 15 soil simulations. **b** The contig distribution within 15 clinical simulations

After simulating, the reads were perfectly binned by sequence origin to limit the creation of chimeric contigs. Chimeric contigs are assembly errors when reads from different organisms are assembled together resulting in a shorter fragmented assembly or taxonomic misclassification downstream. The decision to bin prior to assembly was to allow for genera labeled contigs in order to explore false positive and recall rates of bacteria and phage, respectively. The perfect bins were assembled using metaSpades (version 3.11.1) and only contigs of length 1KB or greater were retained [41]. The relative abundance of bacteria genera in the simulations are shown in Fig. 2.

### Integrated prophage identification

Integrated prophage elements were identified in complete bacterial genomes using VirSorter prior to read simulations [28]. Integrated prophages were selected for downstream processing if assigned as category 4, the highest confidence category for prophages within VirSorter [28]. A nucleotide BLAST database was created with the identified prophage elements. After read simulation and assembly, bacterial contigs were identified as prophages using a BLASTn search against the prophage database with a bitscore greater than 1000 and a percent identity greater than 95%. Additional file 2: Figure S1 shows the genera distribution of the identified prophage elements separated by read distribution and sampling site.

### Tools used in simulation study

**Table 1:** Tools used in viral identification benchmarking study

Tool	Last updated	Target	Viral homology matching	Compositional protein features	Machine learning classification	Programming skills required
*VirSorter*	2015	Virus	Yes	Yes	No	No
*VirSorter2*	2020	Virus	Yes	Yes	Yes	No
*VirFinder*	2017	Virus	No	No	Yes	Yes
*DeepVirFinder*	2020	Virus	No	No	Yes	Yes
*Vibrant*	2020	Virus	Yes	No	Yes	Yes
*MetaPhinder*	2016	Phage	Yes	No	No	Yes
*Earth Virome*	2020	Virus	Yes	No	No	Yes
*Phybrid*	2020	Phage	No	Yes	Yes	Yes
*Kraken2+VirKraken*	2020	Virus	Yes	No	No	Yes

The tools used in the study shown in Table 1 were tested on their performance to identify viral elements from assembled contigs in the simulations. The tools used in this study to identify viral contigs were Vibrant (Version 1.2.0), VirSorter, VirSorter2, VirFinder, DeepVirFinder, MetaPhinder, Kraken 2, Phybrid, and a BLAST search using identified proteins from the Earth Virome Pipeline [15, 16, 19, 28].

Any VirSorter predictions that were classified to the lowest confidence category were removed via evidence by the tool developers [28]. VirFinder and DeepVirFinder assign a probability value and any contigs that had a value less than 0.01 were classified as viral. A diamond blast database was created with the viral proteins from the Earth Virome Pipeline [18, 42]. Proteins from the simulation contigs were predicted using Prodigal and searched for viral homology using diamond BLASTp against proteins from the Earth Virome Pipeline with matches retained that had a bit score greater than 100 and an e-value less than 1e−05 [31]. Contigs with more than one hit were classified as viral. MetaPhinder, Phybrid, Phybrid Proteins, and Vibrant were run with default parameters [13, 15]. Double-stranded DNA phages and single stranded DNA viruses were selected with the groups parameter of VirSorter2 as described by the authors [16]. Kraken 2 was run with default parameters using the minikraken database from March 2020 [21]. The resulting Kraken 2 report was parsed for viral reads using VirKraken (Version 0.0.5).

### Tool performance scoring

The structure of the simulation allowed for each contig to possess a true origin label. These labels were used to identify the performance of the tools to identify viral elements in the simulations. The performance was measured by precision, recall, and F1 score. Prophages were considered viral in this study and an additional analysis of tool performance on prophage identification was performed. The performance measures were used in a simulation performance ranking system to determine the best performing tool across different scenarios. The performance of each tool was ranked within each condition with 1 representing the best performing tool. The highest-ranking value (worst performing tool) changes as some tools were unable to properly calculate a score. This occurs when a tool did not predict any viral element in a simulation.

In addition to overall performance, tool performance is evaluated at four discretized contig lengths: 1 KB–2.5 KB, 2.5 KB–5 KB, 5 KB–10 KB, 10 KB+. The recall of the tools to identify viral elements by genera was used to determine any systematic biases for or against specific viral groups. Visualizations of scoring metrics were performed in Python using a combination of Matplotlib (version 2.2.3) and Seaborn (version 0.9.0) plotting software [43, 44]. Kruskal-Wallace nonparametric testing was performed to determine if the scoring values arose from the same distributions.

## Results

### Overall tool performance

**Fig. 3:**
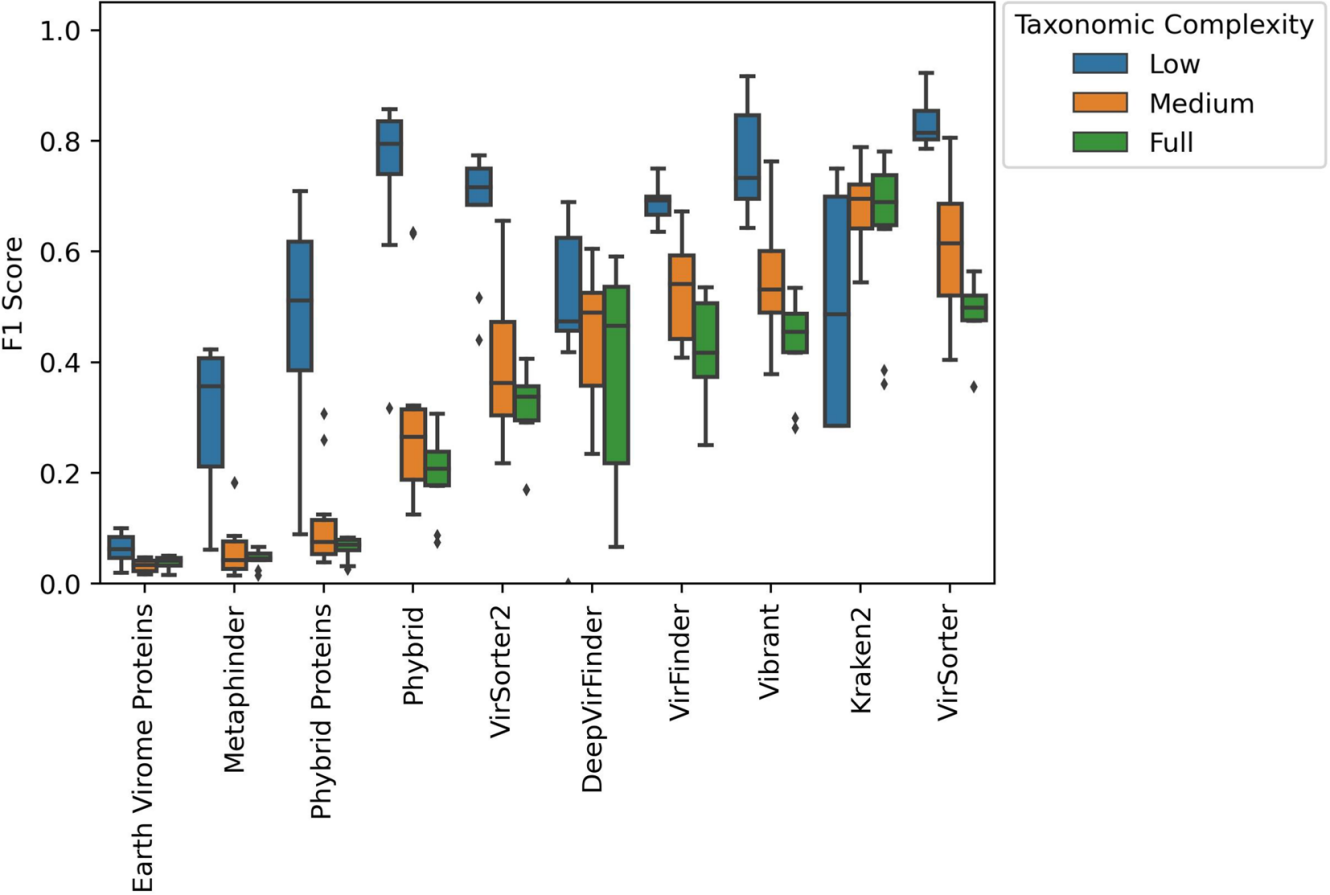
F1 scores of tools by taxonomic conditions. Dodge boxplots by taxonomic complexity arranged by average F1 performance with the best performing tools on the right side of the x axis

The F1 performance across different read simulation conditions was not significantly different (H = 4.02, *p* = 0.404, Kruskal–Wallis). The F1 performance was significantly different by taxonomic complexity with better tool performance in lower complexity simulations relative to both medium and full complexity simulations (H = 47.65, *p* = 4.50e−11, Kruskal–Wallis). The F1 performance, as well as precision and recall, of longer contigs specially the 10 KB+ bin was higher relative to other contig length bins (H = 275.7, *p* = 1.82e−59, Kruskal–Wallis). Table 2 contains the mean performance of the tools and the average ranking across the 30 simulations. The F1 performance of the tools in the simulation discretized by taxonomic complexity is shown in Fig. 3.

**Table 2:** Average performance and simulation rankings of tools at identifying phage

Tool	F1 rank	Precision rank	Recall rank	Average F1	Average precision	Average recall
*VirSorter*	**2.10**	3.10	6.40	**0.636**	0.640	0.658
*Kraken2*	2.93	**1.07**	7.80	0.609	**0.962**	0.467
*Vibrant*	3.52	4.10	7.26	0.560	0.573	0.598
*VirFinder*	3.93	2.30	9.32	0.548	0.717	0.450
*DeepVirFinder*	5.04	5.38	7.90	0.432	0.392	0.496
*VirSorter2*	5.27	5.93	3.10	0.463	0.341	0.797
*Phybrid*	5.40	6.00	3.60	0.413	0.317	0.755
*Phybrid Proteins*	7.47	7.70	4.63	0.142	0.213	0.717
*MetaPhinder*	8.73	8.67	2.83	0.082	0.138	0.842
*Earth Virome*	9.73	9.73	**1.78**	0.023	0.044	**0.872**

Score of best performing tool is bolded in each column

Kraken2 led both average precision and precision rank. In this study, the BLASTp search of proteins from the Earth Virome Pipeline performed best in both recall and recall rank. The tool with the highest average F1 score and best F1 rank was VirSorter. VirSorter was also the tool used to perform prophage identification. This may provide VirSorter with an advantage over other tools in prophage identification.

### Prophage identification performance

The prophage performance of the low complexity simulations are removed due to the presence of only a single prophage contig in all 10 simulations. The F1 performance of the tools to identify prophage in 20 medium and high complexity simulations is shown in Table 3.

**Table 3:** Average performance and simulation rankings of tools at identifying prophage

Tool	F1 rank	Precision rank	Recall rank	Prophage F1	Prophage precision	Prophage recall
*Vibrant*	**1.15**	1.95	7.45	**0.169**	0.146	0.231
*VirSorter*	2.11	**1.66**	8.76	0.147	0.144	0.164
*VirSorter2*	2.70	3.40	4.70	0.117	0.0685	0.453
*Phybrid*	3.95	4.45	7.13	0.0446	0.0269	0.252
*Phybrid Proteins*	5.20	5.50	6.05	0.0188	0.00978	0.342
*Kraken2*	6.70	1.85	9.90	0.0169	**0.172**	0.00896
*MetaPhinder*	6.65	6.85	2.90	0.0152	0.00776	0.588
*Earth Virome*	6.85	7.00	**1.60**	0.0117	0.00588	**0.728**
*VirFinder*	8.88	8.88	2.73	0.00725	0.00365	0.705
*DeepVirFinder*	8.79	9.03	2.79	0.00647	0.00325	0.637

Score of best performing tool is bolded in each column

### Tool performance by contig length

**Fig. 4:**
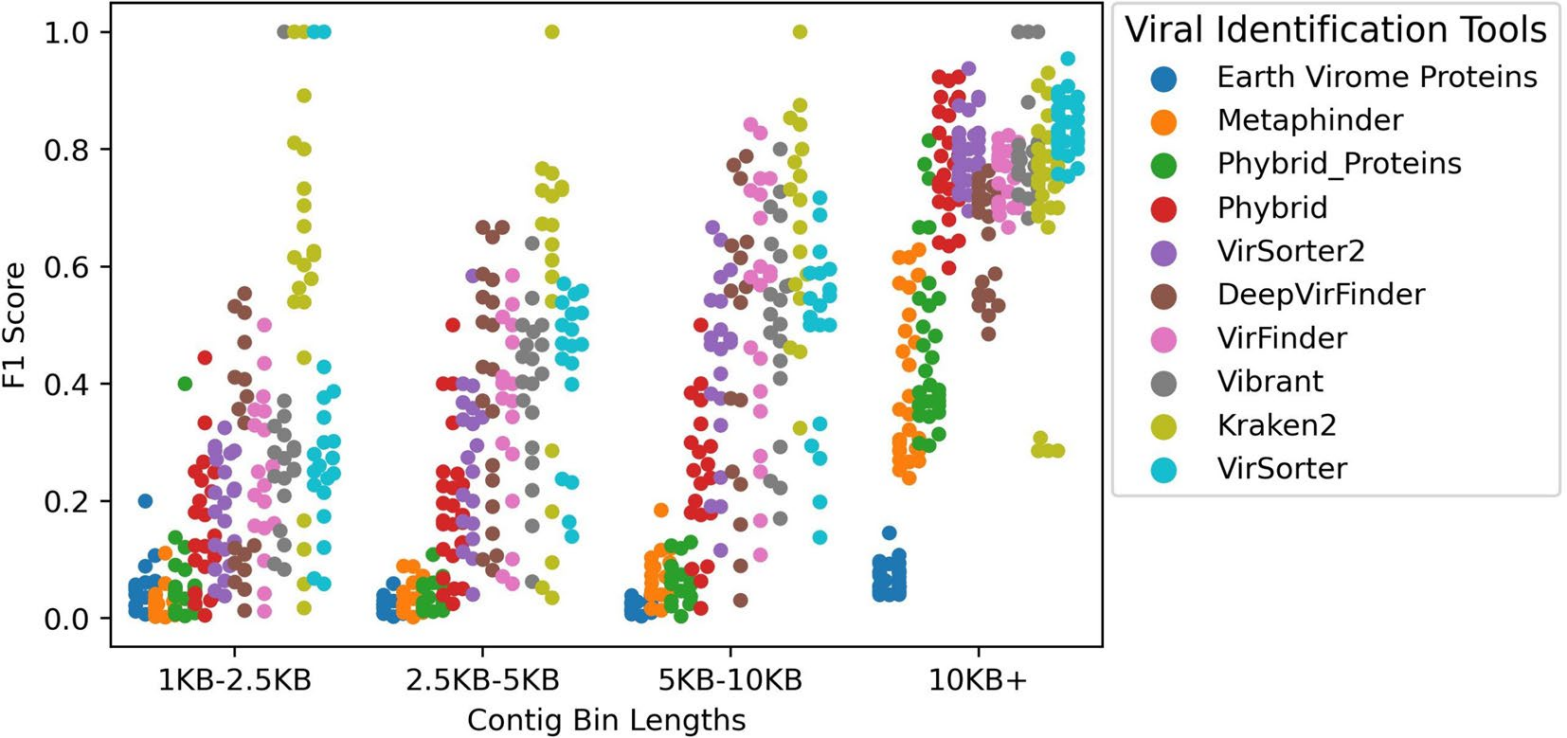
F1 scores of tools across contig length bins in all simulations. The average F1 performance of all tools increases as the bin representing contig lengths increases. All thirty simulations are included as part of this figure, however in some simulations, predicted viral contigs of a specific length are absent. This may cause some tools to have more data points than others

As the length of the contigs increased, the performance of the tools improved. Mean contig length of the simulations was affected by the taxonomic complexity in this study as shown in Additional file 2: Figure S2. Figure 4 demonstrates the F1 performance of each tool within defined contig length bins. If the F1 score of a tool was 0, the record was removed as some lower complexity simulations lacked shorter contiguous sequences.

### Viral recall by host genera

**Fig. 5:**
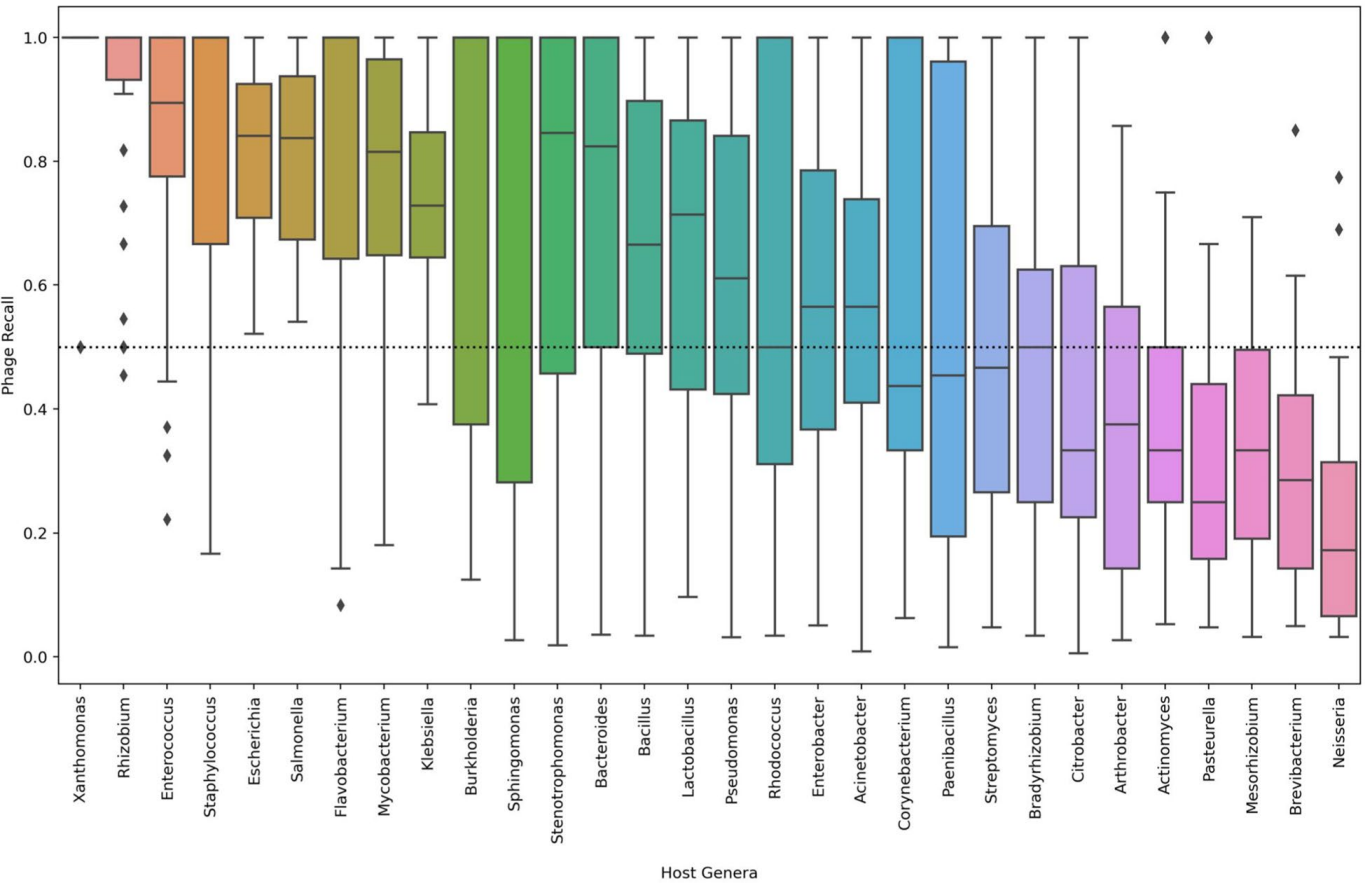
Viral recall by host genera in medium and full complexity simulations. The 30 host genera of phage are listed in order of mean recall along the x-axis. The dotted line in the figure demarcates 0.5 recall

Recall scores of viral elements from the medium and full distributions were calculated across 30 host genera. Recall was only retained if greater than 0 to prevent the absence of a phage host genera by niche. **Figure** 5 shows the recall of viral contigs by host genera across all tools. The viral host genera with the best recall was *Xanthomonas*, however, phage with *Xanthomonas* as a host were not well represented in the data set. Phage known to infect *Enterococcus* achieved an average recall over 0.83 across all tools. DeepVirFinder performed the best at identifying phage known to infect *Enterococcus* with an average recall rate of 0.97. *Neisseria* prophage sequences had the lowest average recall performance across all tools (0.23), with only 7 tools correctly predicting at least one *Neisseria* prophage contig. While multiple contigs were derived only a single *Neisseria* prophage was included in this study and that may be affecting tool performance. The BLASTp search using the proteins from the Earth Virome Pipeline performed the best at identifying this elusive prophage (0.68) and the next best tool was MetaPhinder with a recall rate of 0.24.

### False positive genera

In addition to the recall rate of viral elements by host genera, the percent of genera associated with bacterial false positives was calculated for each tool in medium and full complexity simulations. Bacterial genera that represent more than one third of false positives of a tool in a simulation were retained. Eleven genera were represented with *Streptomyces* present in 9 of 10 tools. Additionally, *Citrobacter* and *Pseudomonas* were major false positive genera in more than 5 tools. Additional file 2: Figure S3 shows the genera of false positives that represent more than 33% by tool.

## Discussion

This study benchmarked and evaluated the ability of nine viral classification tools to identify viral and prophage elements within shotgun metagenomics. The study consisted of 30 Illumina MiSeq simulations across two communities, five read abundance distributions, and three taxonomic levels. The performance of the tools was consistent across read distributions (H = 4.02, *p* = 0.404, Kruskal–Wallis), whereas, the average performance increased with a reduction in taxonomic complexity (H = 47.65, *p* = 4.50e−11, Kruskal-Wallis). Lower taxonomic complexity was associated with longer contig lengths in the assemblies (Additional file 2: Figure S2) and longer contigs were associated with improved overall performance.

The differences between performance scores suggests the selection of a tool may depend upon the desired application. VirSorter scored the highest average F1 score and had the best F1 ranking across all the simulations. Kraken2 may be the ideal tool when minimizing the number of false positives. The BLASTp search using the Earth Virome proteins had the best recall; however, the application of this tool is not meant for traditional viral identification due to the large false positive rate. In this study, the application of a BLASTp homology search using the Earth Virome protein set was performed rather than an extensive domain homology search due to run-time concerns. The Earth Virome protein set was derived from an iterative viral protein domain search and may include many unknown proteins that may not truly be derived from viral sources [18]. Even with this expanded protein set relative to known viral proteins from RefSeq, the broad homology search space still failed to capture all viral derived contigs demonstrating the difficulty of viral identification within metagenomes.

A limitation of this study is the absence of eukaryotic sequences from the simulations. The presence of eukaryotic sequences may further reduce the precision of the tools to identify viruses. Ponsero and Hurwitz described the high false positive rate of k-mer based models on eukaryotic sequences in aquatic metagenomes [45]. This high false positive rate is likely a result of the absence of eukaryotic sequences in the training data of the tool [19]. Machine learning tools without eukaryotic sequences in the training set may produce additional false positives. Any machine learning tool with gaps of eukaroytic or novel viral sequences in the training data may produce errors in viral classification on real metagenomic data.

Prophage identification in metagenomics is a difficult problem as many integrated viral elements are degraded in bacterial hosts to drive evolution [46]. As such, remnants of prophage particles are scattered across bacterial genomes and viral genes can be mistakenly attributed as bacterial in origin. Many tools to identify prophages in whole genome experiments fail to generalize to metagenomics due to fragmentation that breaks down traditional viral enrichment measurements. The decision to select the highest confidence prophage predictions using VirSorter from the complete genomes prior to simulation may have provided VirSorter with an added performance boost. Vibrant had the highest average F1 score and best F1 ranking at identifying prophages across all 20 simulations. Kraken 2 had the highest average precision and VirSorter had the best precision ranking. The Earth Virome proteins exceled at recall; however, the next best tools were VirFinder and DeepVirFinder. VirFinder and DeepVirFinder like many other tools that perform well with prophage recall have a high false positive rate.

The performance of all tools would increase with an additional step of removing known bacterial contigs. One approach is to search for genes unique to bacteria and archaea, the 16S rRNA. 16S rRNA profiles from RFAM can be applied to the RNA domain search tool, Infernal, to remove contigs with known bacterial genes [47, 48]. This approach may impact the recovery of prophage contigs if the integration site of the virus was near a 16S rRNA.

Viral identification tools performed well at identifying phages known to infect genera such as *Enterococcus* (0.83), *Mycobacterium* (0.77), and *Salmonella* (0.81). The performance of the tools to identify phages that infect genera such as *Neisseria* (0.23), *Brevibacterium* (0.30), and *Mesorhizobium* (0.33) dropped substantially. Detecting the presence of *Neisseria* phage and prophage may be important for a diagnostic of invasive meningococcal disease as prophage-like elements are commonly found throughout the *Neisseria* genera [49]. The results of the tools on individual genera are meant to demonstrate the variability of tool performance on different genera of phage. Phage genera in this study are defined by host range. Phage host range is poorly understood and there exists sampling bias towards phages affecting more well studied bacterial pathogens. The results of this study seemed to imply that sampling bias of phage genera in public databases may be affecting many overall tool performances. *Mycobacterium* phages and *Enterococcus* phage are the most abundant phages in public databases. The results of phage genera performance should not be over-interpreted in this study as the number of unique phages known to affect a bacterial genera are not uniformly distributed as seen in Additional file 2: Tables S1 and S2.

The performance of Phybrid including the nucleotide features showed improvement over the gene content features alone. The precision of Phybrid dramatically improved with contigs over 10KB, however, smaller bins were plagued with many false positives. Integrated prophages added to the viral class in the training data represented 28.3% of the total viral genomes. Prophages are commonly degraded in bacterial hosts to drive evolution [46], therefore degraded viral elements in bacterial contigs with similar nucleotide structures as the complete prophages may be misclassified. In addition, the use of k-mer profiles for smaller contig classification created sparse data sets, which may have led to overfitting.

The performance of the tools presented needs to be weighted with the computational cost to run each tool. This study was performed on a shared high performance computing cluster and individual tool performance and memory requirements were not captured on an isolated node. However, the mechanism of viral identification can be used infer the relative time and memory consumption of the tools. The fastest tool in this study was Kraken2, which uses discriminatory k-mers to compare against a pre-computed hash table. The amount of memory needed to build the full hash table may be a drawback against using Kraken2 on a personal machine. The tool, Vibrant, uses protein features derived from multiple HMM searches. As a result of a large domain space, this tool ran for a significantly longer amount of time (1 week for full complexity simulations) relative to the other tools on the shared compute cluster.

This study benchmarked and compared the performance of viral identification tools in metagenomics. The viral identification performance measures, in conjunction with the genera and prophage recall, highlights the advantages and challenges of using specific viral identification tools, and can be used as a guide to assist the selection of tools for subsequent research.

## Conclusion

In summary, we tested the performance of nine viral identification tools on 30 simulated metagenomes. The underlying read distribution has little impact on average tool performance. Increasing contig length and decreasing taxonomic complexity improved the average performance of the tools. Vibrant performed the best at the identification of prophages in metagenomics. Overall, the tool that averaged the best F1 score was VirSorter, while Kraken2 lead all other tools in precision. The results of these simulations should provide researchers with a guide to selecting the appropriate tool for their own viral identification research.

## Supplementary Information

**Additional file 1**. Supplemental figures and tables referenced in the article.

**Additional file 2**. Captions of supplemental figures and tables referenced in the article. 

## Data Availability

All scripts used to derive the figures and additional preprocessing workflows are available on the Strong Lab GitHub at https://github.com/Strong-Lab/Viral_Classification_in_Metagenomics. Phybrid is available on the Strong Lab GitHub at https://github.com/Strong-Lab/Phybrid. VirKraken, the Kraken2 extension used in this study is available on the Strong Lab GitHub at https://github.com/Strong-Lab/VirKraken and on PyPI. The fasta files of the simulations are found at https://tinyurl.com/fastavm.

## Abbreviations

contig: Contiguous sequence
BAM: Bacteriophage adherence to mucus
IBD: Inflammatory bowel disease
VLP: Viral like particles
pVOG: Prokaryotic virus orthologous group
HMM: Hidden Markov model
EMP: Earth Microbiome Project
KB: Kilo-basepairs
