# Supplementary material for: Simulation study and comparative evaluation of viral contiguous sequence identification tools
Source: BMC Bioinformatics. 2021 Jun 16;22:329. doi: 10.1186/s12859-021-04242-0 (PMC8207588; doi:10.1186/s12859-021-04242-0)
Supplement: Supplementary file 1 — Additional file 1. Supplemental figures and tables referenced in the article. [file 12859_2021_4242_MOESM1_ESM.pdf]

Simulation Study and Comparative Evaluation of Viral Contiguous Sequence Identification Tools

Cody Glickman, Jo Hendrix, Michael Strong

1 Supplemental Figures

Supplemental Figure 1: Relative abundance of genera in prophage contigs

The distribution of prophage host genera in each niche separated by underlying read distributions.

Supplemental Figure 2: Log-normalized contig lengths by simulation complexity

Lengths of contiguous sequences across simulations by complexity. Lower complexity simulations were associated with a higher average contig length than more complex simulations.

Supplemental Figure 3: Abundant false positive genera in medium and full complexity simulations

The distribution of 12 bacterial genera that comprise at least 1/3 of all false positives by a tool in the medium or full complexity simulations. The most abundant genera represented in this figure is *Streptomyces*, which is also one of the most abundant genera in both clinical and soil distributions.

2 Supplemental Tables

Supplementary Table 1: Genera distributions of soil microbe simulations by taxonomic level and kingdom.

These tables show only the top 6 genera in each condition of the clinical simulations. Bacterial genera are shown on the left and the host genera representing phage genomes are shown on the right.

Supplementary Table 2: Genera distributions of clinical microbe simulations by taxonomic level and kingdom.

These tables show only the top 6 genera in each condition of the clinical simulations. Bacterial genera are shown on the left and the host genera representing phage genomes are shown on the right.
