# Supplementary material for: Simulation study and comparative evaluation of viral contiguous sequence identification tools
Source: BMC Bioinformatics. 2021 Jun 16;22:329. doi: 10.1186/s12859-021-04242-0 (PMC8207588; doi:10.1186/s12859-021-04242-0)
Supplement: Supplementary file 2 — Additional file 2. Captions of supplemental figures and tables referenced in the article. [file 12859_2021_4242_MOESM2_ESM.pptx]

## Slide 1
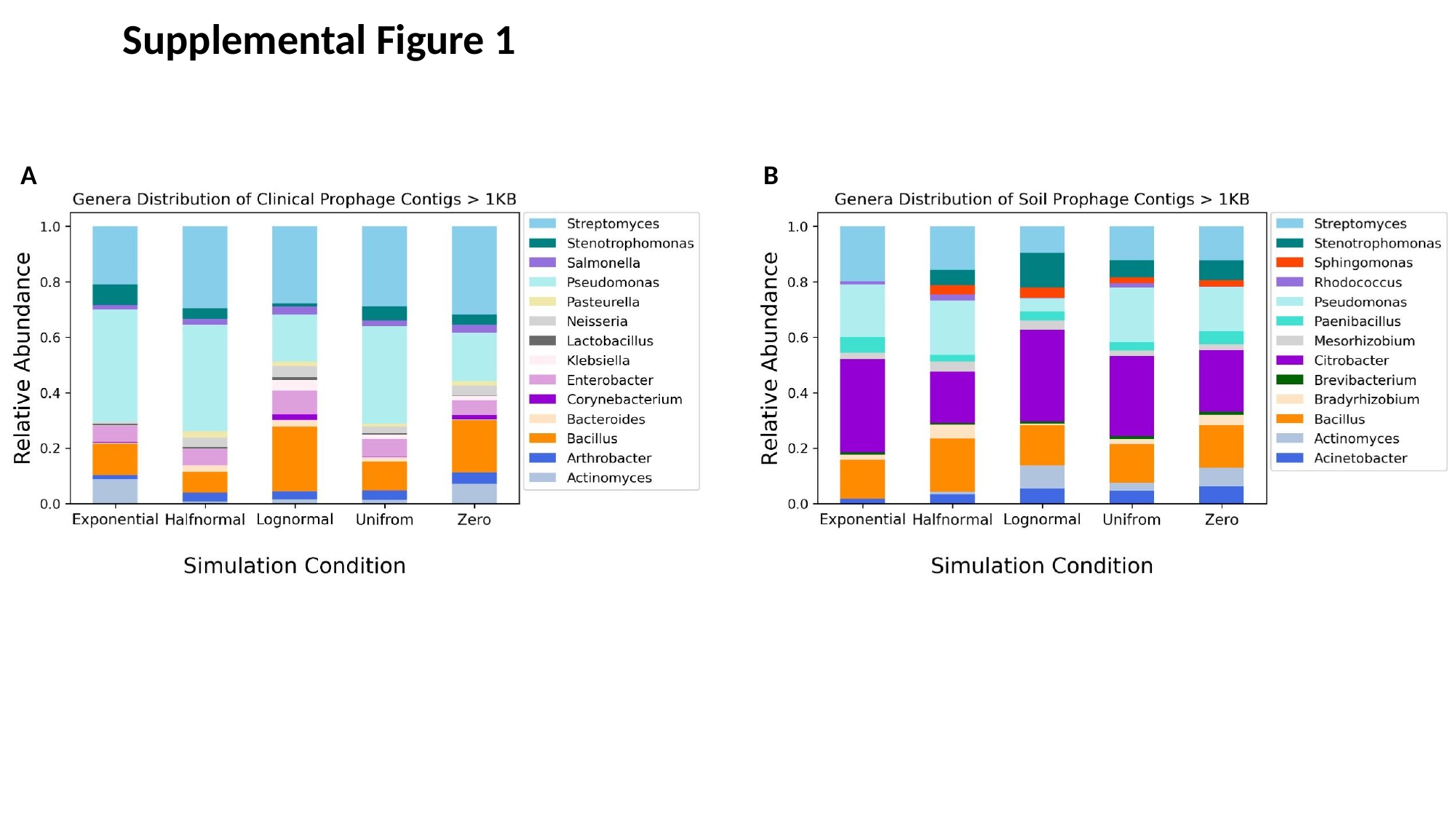

Supplemental Figure 1
A
B

## Slide 2
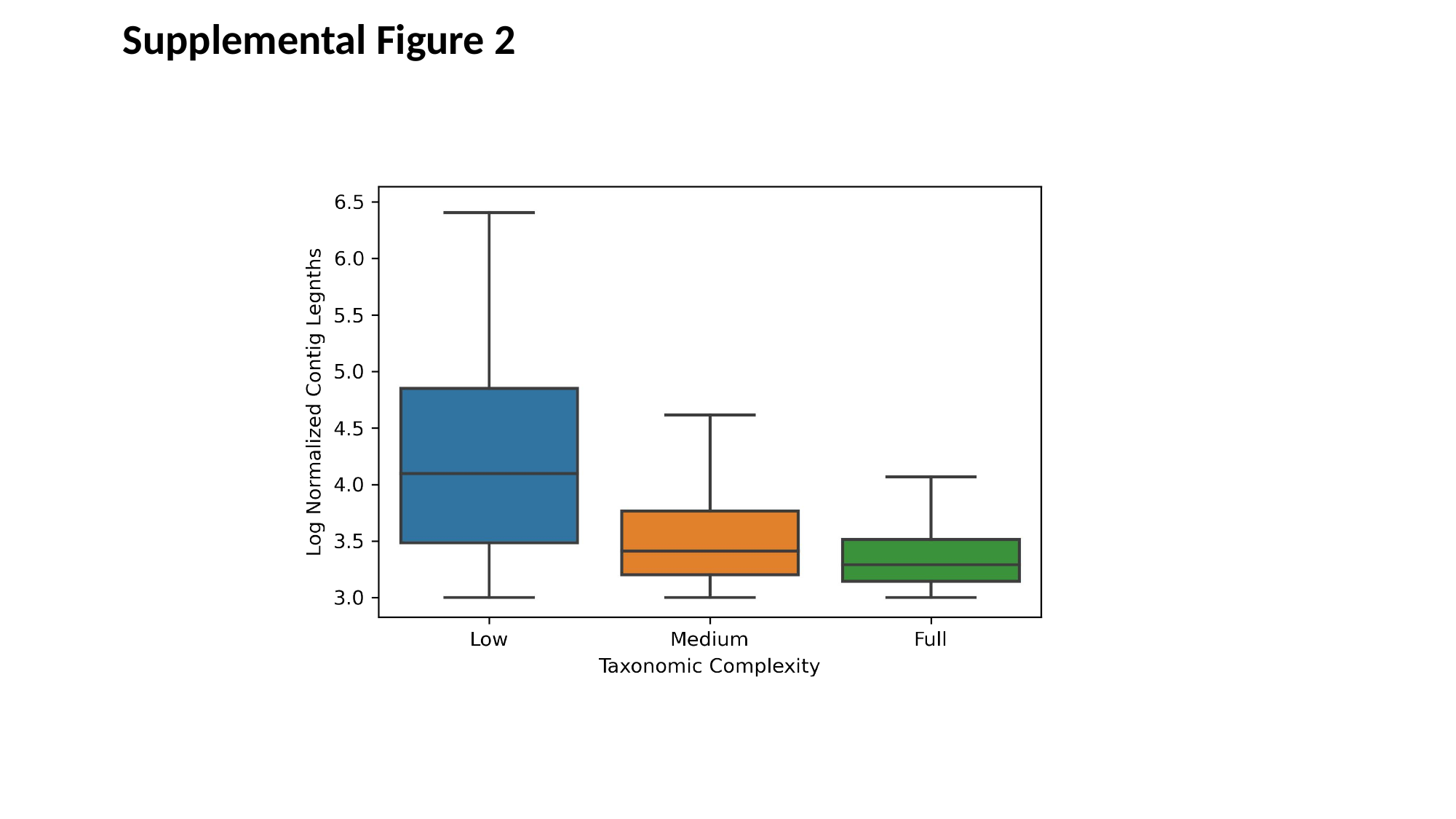

Supplemental Figure 2

## Slide 3
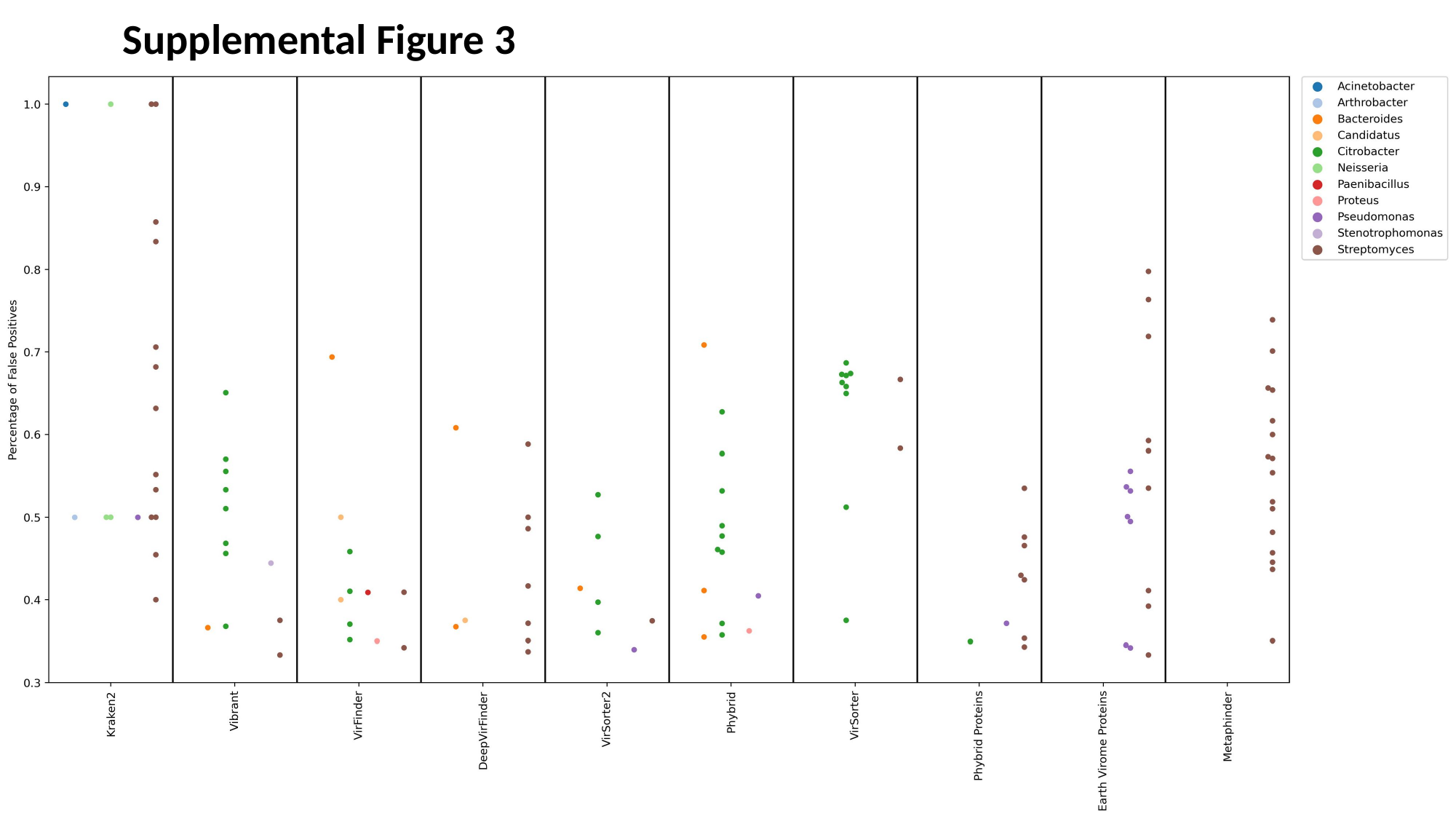

Supplemental Figure 3

## Slide 4
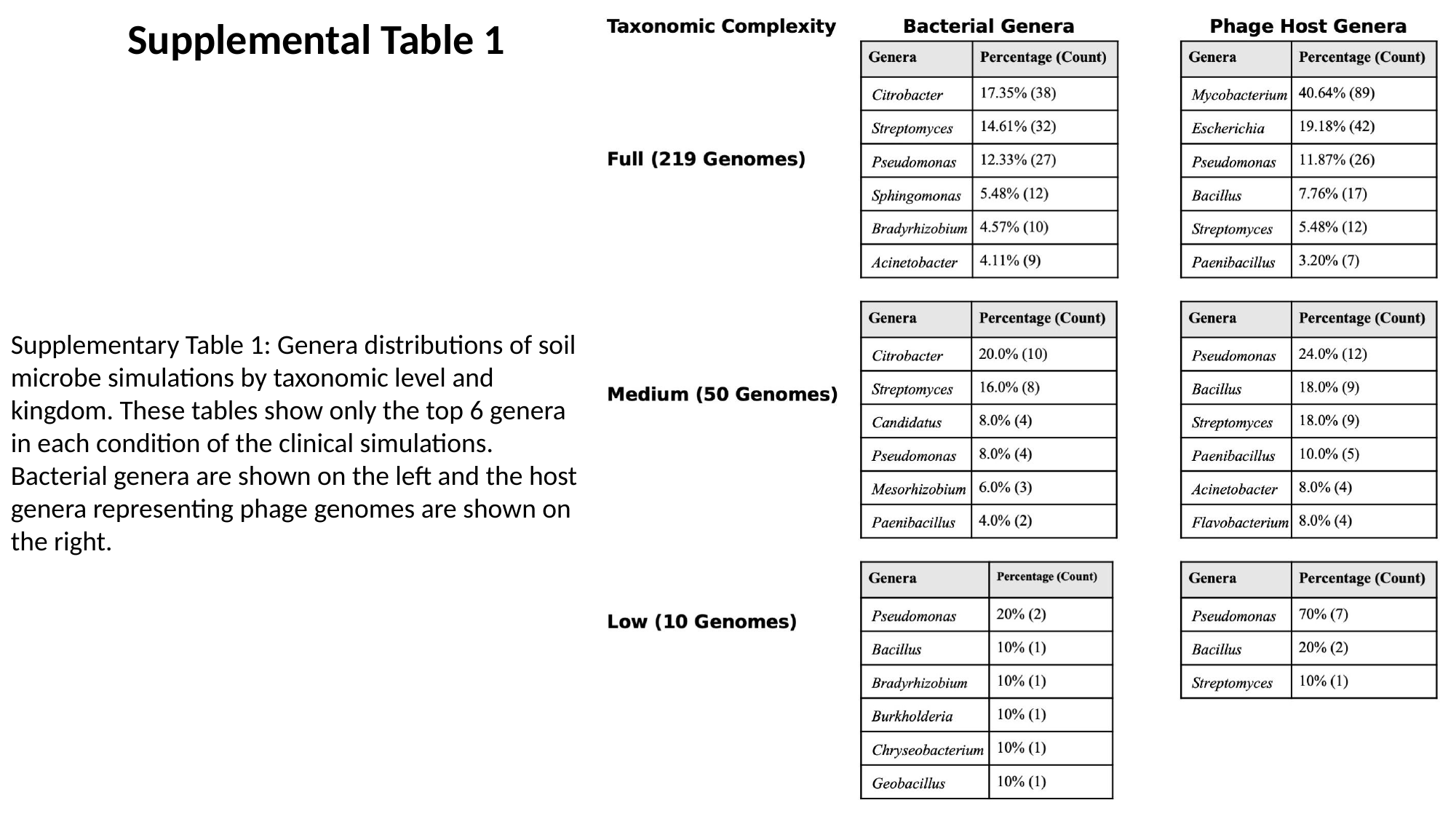

Supplemental Table 1
Supplementary Table 1: Genera distributions of soil microbe simulations by taxonomic level and kingdom. These tables show only the top 6 genera in each condition of the clinical simulations. Bacterial genera are shown on the left and the host genera representing phage genomes are shown on the right.

## Slide 5
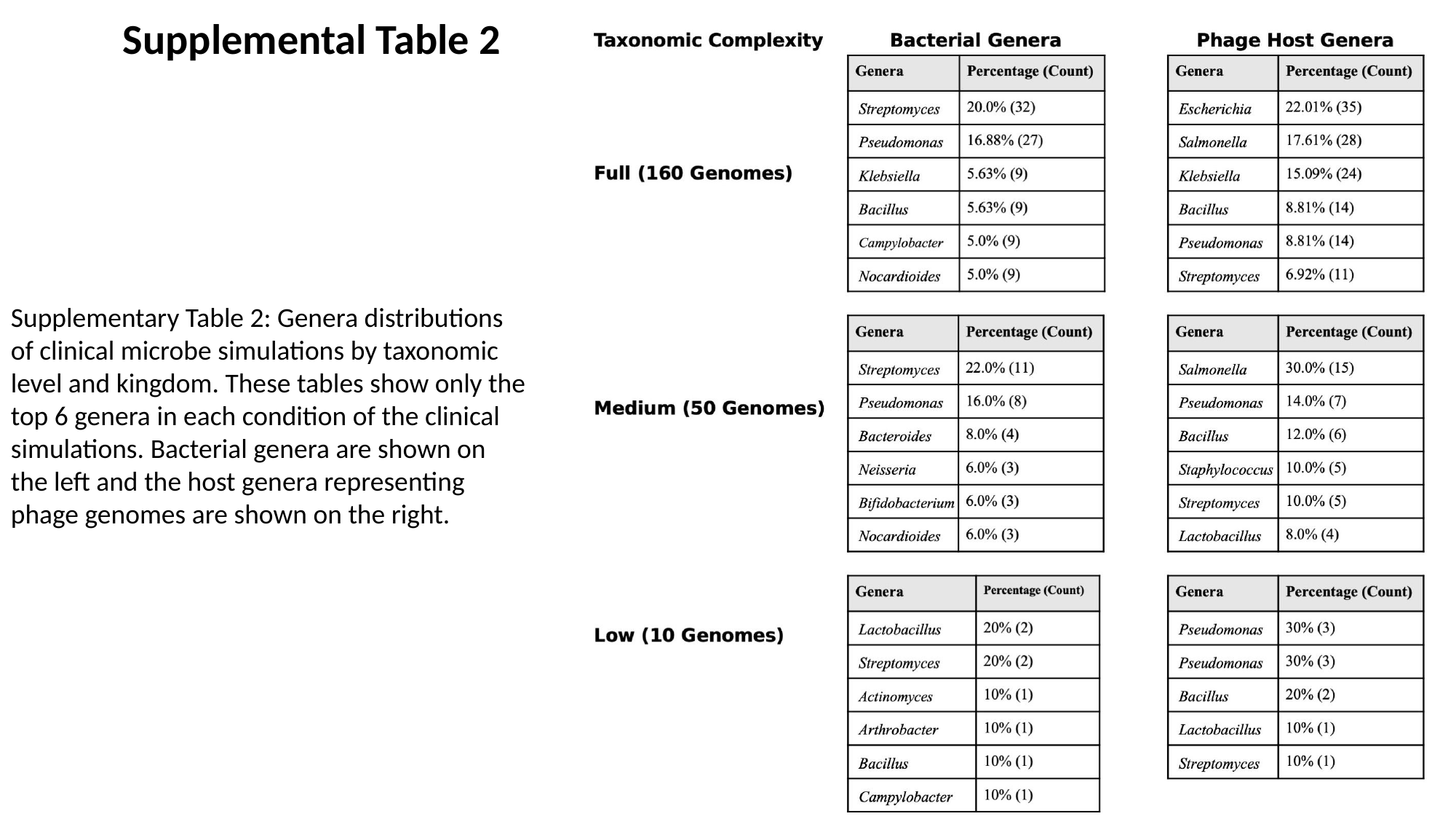

Supplemental Table 2
Supplementary Table 2: Genera distributions of clinical microbe simulations by taxonomic level and kingdom. These tables show only the top 6 genera in each condition of the clinical simulations. Bacterial genera are shown on the left and the host genera representing phage genomes are shown on the right.
